# Acute tryptophan depletion in accordance with body weight: influx of amino acids across the blood–brain barrier

**DOI:** 10.1007/s00702-012-0793-z

**Published:** 2012-05-24

**Authors:** V. L. S. Dingerkus, T. J. Gaber, K. Helmbold, S. Bubenzer, A. Eisert, C. L. Sánchez, F. D. Zepf

**Affiliations:** 1 Department of Child and Adolescent Psychiatry, Psychosomatics and Psychotherapy, RWTH Aachen University, Neuenhofer Weg 21, 52074 Aachen, Germany; 2 JARA Translational Brain Medicine, Aachen and Jülich, Germany; 3 Department of Pharmacy, RWTH Aachen University, Aachen, Germany; 4 Institute for Neuroscience and Medicine (INM), Jülich Research Centre, Jülich, Germany

**Keywords:** Acute tryptophan depletion, Serotonin, Tryptophan, Influx rates, Blood–brain barrier, Moja-De

## Abstract

Acute tryptophan depletion (ATD) is a method of reducing central nervous serotonin (5-HT) synthesis in humans by administering an amino acid (AA) beverage lacking in tryptophan (TRP), the physiological precursor of 5-HT. However, to date, the use of conventional ATD protocols in children and adolescents was limited due to frequently observed side effects (e.g., vomiting and nausea). This study investigated the effects of diminished central nervous system 5-HT synthesis on plasma concentrations of relevant AAs and TRP influx into the brain in 24 healthy young adults using the ATD procedure Moja-De, a test protocol that has been used in preliminary research in youths. Twenty-four healthy participants received ATD and a TRP-balanced amino acid load (BAL) using a randomized double-blind within-subject crossover design. Plasma concentrations of the relevant AAs that compete with TRP on the same transport system were assessed at baseline and 90, 180, and 240 min after ATD/BAL intake. TRP influx across the blood–brain barrier was calculated using Michaelis–Menten kinetics with a correction for multiple substrate competition, indicating a significant decrease in TRP influx into the central nervous system under Moja-De. ATD Moja-De decreased TRP influx into the brain and central nervous system 5-HT synthesis safely and effectively and was well tolerated, allowing it to be used in children and adolescents. Future research into other secondary, compensatory effects induced by ATD in patients with neuropsychiatric disorders and healthy populations is needed. ATD Moja-De allows this type of research with a focus on a developmental viewpoint.

**Electronic supplementary material:**

The online version of this article (doi:10.1007/s00702-012-0793-z) contains supplementary material, which is available to authorized users.

## Introduction

The neurotransmitter serotonin (5-HT) plays an important role in many neuropsychiatric disorders and behavioral phenotypes, in particular, affective disorders, eating and attention disorders along with their changed cognitive processes, aggressive behavior and impulsivity. To date, several methods have been used to probe the effects of central nervous 5-HT in the described patient populations and in healthy subjects. Along with the study of genetic differences between healthy controls and patients with various neuropsychiatric disorders, several strategies have been adopted to study 5-HT function, such as the assessment of the 5-HT metabolite 5-HIAA in cerebrospinal fluid, the prolactin (PRL) response to fenfluramine (FEN) administration, and the uptake of 5-HT in platelets. However, studying central nervous 5-HT function in children and adolescents is difficult, particularly because many previously used methods are rather invasive (i.e., such as the assessment of 5-HIAA in CSF) or cannot be undertaken because of cardiac risks, especially valvular heart disease [PRL–FEN response, (Connolly et al. 1997; Graham and Green 1997; Li et al. 1999)]. In addition, the administration of SSRIs to youths as a way to probe central nervous 5-HT function by enhancing the availability of 5-HT is limited in terms of randomized controlled trials, in particular when healthy controls are involved.

Acute tryptophan depletion (ATD) is one possible way to achieve a short-term serotonergic dysfunction in the central nervous system in humans. The administration of an amino acid beverage that lacks TRP, the physiological amino acid precursor of 5-HT, diminishes its uptake into the brain. ATD occurs because all the relevant amino acids in the beverage use the same active large neutral amino acid (LNAA) transport system (L-1) in the capillary cell plasma membrane to pass the blood–brain barrier. The L-transport system was localized in different kinds of cells in the human body. However, the L-1-system at the capillary endothelial cells of the blood–brain barrier differs from the L-system found in other human cells (as for example in the gastrointestinal tractus, hepatic and renal tissue) because of its 100- to 1,000-fold higher affinity for the outlined amino acids (Oldendorf and Szabo 1976; Kewitz 2002). In past research the sum for the unidirectional influx of all LNAAs was calculated with 50 nmol/min/g brain tissue (Oldendorf and Szabo 1976; Smith et al. 1987a, b). As was demonstrated by previous work, the uptake of amino acids using L-1 at the blood–brain barrier marks a facilitated diffusion of amino acids into the brain and follows Michaelis–Menten kinetics with competitive substrate inhibition (Smith et al. 1987a, b; see also Kewitz 2002). In order to determine influx-properties and affinity constants of different amino acids in order to be able to calculate influx rates several in vitro and in vivo methods were developed (e.g., in vitro measurements with isolated capillary endothelial cells of the blood–brain barrier, indicator-dilution technique, etc., see Kewitz 2002 and Pardridge 1998). Such information on affinity constants and influx rates of different amino acids is of particular importance in order to perform quantitative calculations in humans. As outlined by Kewitz (2002), the affinity towards L-1 of different amino acids is correlated with the polarity of the molecules, and unipolar hydrophobic amino acids (such as tryptophan and phenylalaline) were shown to have the highest affinity with respect to L-1 as indexed by their rather low *K*_m_ values (in enzyme kinetics, *K*_m_ values approximate the affinity of a particular enzyme for a specific substrate). For example, a low *K*_m_ value is indicative of a rather high affinity of a particular amino acid for the L-1 transporter, which in turn leads to a faster accomplishment of the maximum rate of conversion (*V*_max_). In contrast to this, a high *K*_m_ value would lead to a slower accomplishment of *V*_max_. As was shown by animal research the uptake of LNAAs into the brain is saturated under physiological conditions, and this process is considered to be independent of sodium (Pardridge 1998; Smith 2000). The underlying concept of ATD, a physiological neurodietary method to lower central nervous 5-HT synthesis in humans, builds on the increase in plasma concentrations of other amino acids as achieved by their dietary administration (often after an overnight fasting period), which in turn impacts the transcapillary influx of TRP over the blood–brain barrier which follows the underlying principles of facilitated diffusion. The administration of an amino acid beverage without TRP causes the competitive antagonism at the L-1 of the consumed competing amino acids (CAAs) with endogenous TRP. Due to these amino acids, the overall influx of TRP across the blood–brain barrier is diminished. As a result of the decreased TRP uptake into the central nervous system (CNS), there is reduced substrate availability for tryptophan hydroxylase 2 (TPH2, which is half-saturated under physiological conditions), the enzyme that limits the rate of synthesis of 5-HT in the CNS. Moreover, amino acid intake stimulates protein synthesis in the liver. This synthesis requires additional TRP from plasma stores and contributes to 5-HT depletion, in addition to the passive diffusion of amino acids across the blood–brain barrier (Zepf 2012).

We recently developed a new ATD protocol, Moja-De, that administers relevant amino acids depending on body weight (Kewitz 2002; Demisch et al. 2002), allowing its use in children and adolescents without the side effects, such as vomiting and nausea, that were frequently observed in adult populations (Zepf et al. 2008b, 2009c). This weight-adapted protocol takes a positive correlation between body weight and plasma TRP into account (Kewitz 2002; Demisch et al. 2002). This particular protocol is well tolerated and can be used in children and adolescents (Stadler et al. 2007; Zepf et al. 2008a, b, c, 2009d); however, to date, no publications have demonstrated a decreased influx of TRP into the brain after Moja-De administration. The present explorative approach administered the ATD Moja-De procedure to healthy young adults to validate the procedure used in this study regarding its impact on TRP influx into the brain and its influence on CNS 5-HT synthesis. We calculated the influx data for TRP entering the brain across the blood–brain barrier under the influence of the ATD Moja-De paradigm versus a balanced amino acid load (BAL) that served as the control condition.

## Methods

### Study design

This study employed a randomized double-blind within-subject repeated-measures design to administer ATD and BAL on different days. The ATD/BAL administration was a within-subject repeated-measures factor. After an overnight protein fast, the participants received the ATD/BAL amino acids in a beverage each morning. Before ATD/BAL intake, a baseline (T0) blood sample, drug screen, and pregnancy tests (in females) were administered. Three additional blood samples were taken 90 min (T1), 180 min (T2), and 270 min (T3) after the ATD/BAL intake each day.

### Sample

The sample consisted of 24 participants aged 21–30 years (12 males and 12 females; mean age = 25.34 ± 2.09 years). The mean weight of the whole sample was 70.54 ± 11.86 kg, the mean BMI was 23.04 ± 1.86 kg/m^2^. The complete demographic data of the study sample with respect to the two genders are provided as Supplementary Online Material. The Ethics Committee of the Faculty of Medicine, RWTH Aachen University, approved the study protocol. The inclusion criteria were good physical and mental health, as assessed by an experienced clinician through an interview. The exclusion criteria were an IQ under 85, the presence of a developmental disorder, schizophrenia, affective disorder, psycho-organic syndrome, drug abuse, somatic disease, the regular use of medication, and pregnancy. Before the study, the participants were screened for psychiatric disorders using a standardized interview [SKIDPIT-light; (Demal 1999)]. All the participants provided oral and written informed consent to participate in the study. The participants were financially compensated after the study.

### Depletion procedure

Prior to study participation, recommendations for a standard breakfast containing no TRP were provided to all participants. All participants confirmed that they had followed these recommendations on each day of testing. Moja-De administers amino acids (AAs) within an aqueous suspension, in which the relevant AA quantities are in accordance with the participants’ body weights (Stadler et al. 2007; Zepf et al. 2008a, b, c, 2009a, b, d; Zepf and Poustka 2008). After an overnight protein fast, some participants received ATD on 1 day and BAL on another day; other participants received these solutions in the reverse order. The AA quantities in Moja-De were as follows (dosage per 10 kg of body weight): l-phenylalanine (PHE 1.32 g), l-leucine (LEU 1.32 g), l-isoleucine (ILE 0.84 g), l-methionine (MET 0.5 g), l-valine (VAL 0.96 g), l-threonine (THR 0.6 g), and l-lysine (LYS 0.96 g). The BAL beverage contained the same AA quantities with an additional 0.7 g of TRP per 10 kg of body weight. Nine participants (37.5 %, six females and three males) received ATD on their first day, and 15 participants (62.5 %, six females and nine males) received ATD on their second day of participation.

### Laboratory assessment

Blood samples were collected for each time point in heparinized and nonheparinized tubes. After collection, they were kept at room temperature for 30 min and then centrifuged at 3,500*g* for 10 min. Subsequently, the remaining serum and plasma were placed in new tubes, and all the tubes were kept at −80 °C until their transportation to the laboratory and analysis. Plasma AA concentrations were assessed using high-pressure liquid chromatography (HPLC) after precolumn derivatization using ortho-phthaldialdehyde (OPA). Albumin-bound TRP was separated from free TRP using an Amicon Ultra-0.5 centrifugal filter at 14,000*g* for 30 min (Merck-Millipore, Darmstadt, Germany), which retains compounds larger than 10 kDa.

### Calculation of the TRP influx into the brain

TRP influx across the blood–brain barrier into the brain is characterized by unidirectional uptake and depends on TRP concentrations and competing LNAAs. The brain capillary LNAA carrier L-1 is the main transport mechanism for LNAAs, and these cannot be synthesized in the CNS (Oldendorf and Szabo 1976; Pardridge 1983; Smith and Stoll 1998). As a consequence, the unidirectional influx rates for the AA uptake from the plasma into the brain in terms of a transcapillary influx following facilitated diffusion at L-1 can be calculated using the Michaelis–Menten equation with a correction for multiple substrate competition (Pardridge 1983; Smith et al. 1987a). As regards the used approach Michaelis–Menten kinetics provide a simple and valid mathematical model to describe the relationship between a substrate and its availability after reaction of the substrate with an enzyme to form a specific product. Briefly, Michaelis–Menten kinetics can describe the conversion of a substrate by an enzymatic mechanism with respect to the availability and concentration of the substrate, and provide an approximation for the reaction rate. The net uptake of TRP over the blood–brain barrier in terms of a transcapillary influx at L-1 builds on two major components: the outlined passive transport of amino acids at L-1 that can be facilitated by integral proteins, and a further proportion that follows passive diffusion (Kewitz 2002). The formula based on Michaelis–Menten kinetics that was used to calculate the influx-rate of tryptophan over the blood–brain barrier (see Kewitz 2002) was

$$ {\text{TRP}}\;{\text{ influx}} = V_{ \max } C/ \left(K_{\text{m}} \left[ 1+ \sum  ({C_{\text{i}} /K_{\text{i}} }) \right] + C \right) + K_{\text{d}} C $$

with *C* = plasma concentration of TRP, *V*_max_ = maximum rate of conversion, *K*_m_ = affinity constant for TRP (Michaelis constant), *C*_i_ = plasma concentration of CAAs, *K*_i_ = affinity constants of CAAs, *K*_d_ = diffusion constant. Of note, *K*_m_ resembles the substrate concentration that is related to the rate of conversion that resembles 50 % of *V*_max_ for the relevant amino acid. In order to perform the above-mentioned calculations, transport constants for different amino acids from previously published studies were adopted and implemented into the mathematical model (Smith et al. 1987a, b; Smith and Stoll 1998).

### Data analyses

The level of statistical significance was set at *p* < 0.05. Because of the exploratory nature of the present investigation, significant *p* values were not subject to alpha adjustments. All the dependent variables were tested for normality using the Kolmogorov–Smirnov goodness of fit test with regard to the full sample, and males and females were also tested independently. Regarding the whole sample, all the variables were normally distributed except THR. Regarding the sexes separately, only the female VAL levels were not normally distributed. Separate repeated-measures analyses of variance (RMANOVAs) investigated the following effects: ATD on TRP, ATD on fTRP plasma concentrations, on the LNAA/TRP ratio, and on the influx of TRP across the blood–brain barrier. Treatment (ATD vs. BAL) and time after intake were the within-participant factors, whereas sex was the between-participants factor. The same within- and between-participant factors were used for separate RMANOVAs to test the effect of ATD on the other AA (i.e., ILEU, LEU, LYS, MET, PHE, THR, TRP, fTRP, TYR, and VAL) concentrations.

## Results

### Effects of Moja-De on amino acid plasma concentrations

**Fig. 1:**
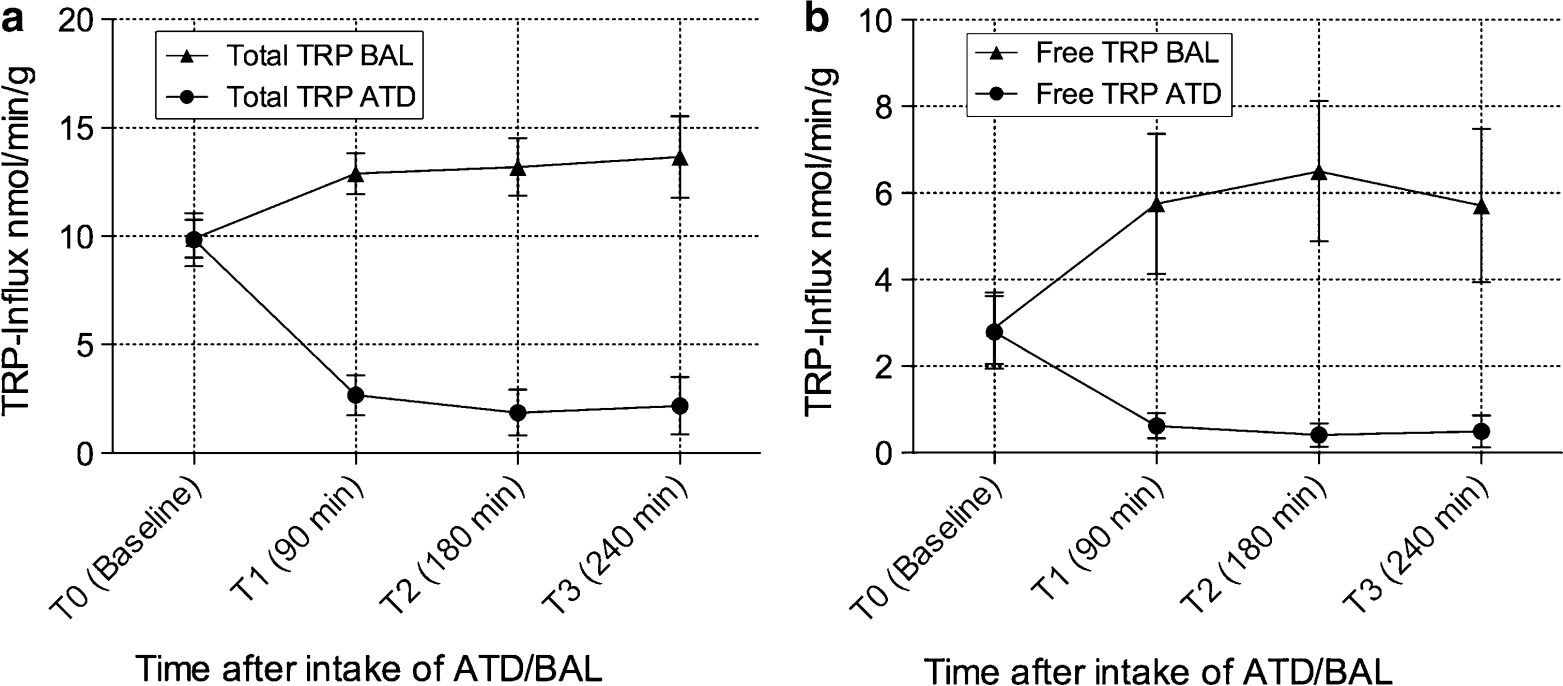
Influx curves (nmol/min/g brain tissue) of the **a** total and **b** free TRP across the blood–brain barrier at the time points T0–T3 after the intake of acute tryptophan depletion (ATD) and a balanced amino acid load (BAL). The data are represented as the mean values ± SD

**Fig. 2:**
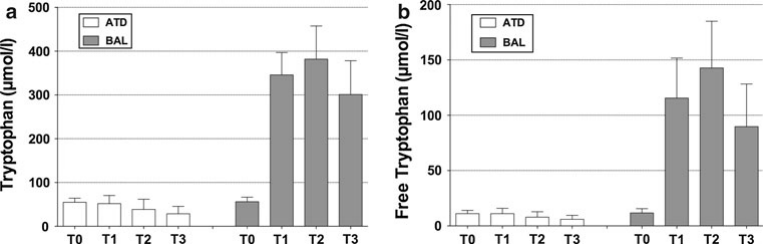
Plasma concentrations (μmol/l) of the **a** total and **b** free TRP at the time points T0–T3 after the intake of acute tryptophan depletion (ATD) and a balanced amino acid load (BAL). The data are represented as the mean values ± SD

Overall ATD Moja-De was well tolerated in the whole sample. The different influx curves for TRP and fTRP are given in Fig. 1a, b. Table 1 provides the degrees of freedom (*df*), *F* values (*F*), and significance (*p* value) of the RMANOVAs for the different AAs. Treatment (ATD vs. BAL, *p* < 0.001–0.023) and (*p* < 0.001–0.001) influenced almost all the AA plasma levels significantly. Figure 2a, b provides all the concentration means and standard deviations of TRP and fTRP for the different treatment conditions (ATD vs. BAL) and time points (T1–T3) in the whole study sample. The data on the different AA concentrations except TRP-derived parameters (TRP, fTRP) are provided as Supplementary Online Material. Only TYR (which was not included in the ATD and BAL beverages) was unaffected by treatment [treatment *F* (1, 92) = 1.076, *p* = 0.311; time *F* (2, 46) = 8.838, *p* = 0.001]. As expected, all the AA concentrations (except TRP) initially increased after ATD administration (see Supplementary Online Material). There were significant effects of both treatment and time, as well as a significant treatment-by-time interaction on the TRP and fTRP levels (see below; Table 1), which indicates that there was a remarkable decrease in TRP and fTRP plasma concentrations after ATD intake for all post-administration time points (see Fig. 2a, b).

**Table 1:** Results including degrees of freedom (*df*), *F* values (*F*), and the significance (*p* value) of different repeated-measures analyses of variance (RMANOVAs) for the amino acids isoleucine (ILE), leucine (LEU), lysine (LYS), methionine (MET), phenylalanine (PHE), threonine (THR), tryptophan (TRP), free tryptophan (fTRP), and tyrosine (TYR)

Amino acid	Within-participant factors/interaction	*df*	*F*	*p* value
ILE	Treatment	1, 92	8.947	0.007
	Time	2, 46	14.980	<0.001
	Treatment × time	2, 92	3.114	0.054
LEU	Treatment	1, 92	6.009	0.023
	Time	2, 46	44.460	<0.001
	Treatment × time	2, 92	1.954	0.154
LYS	Treatment	1, 92	25.449	<0.001
	Time	2, 46	150.016	<0.001
	Treatment × time	2, 92	1.341	0.272
MET	Treatment	1, 92	13.091	0.002
	Time	2, 46	16.319	<0.001
	Treatment × time	2, 92	1.463	0.243
PHE	Treatment	1, 92	6.312	0.020
	Time	2, 46	28.476	<0.001
	Treatment × time	2, 92	1.549	0.224
THR	Treatment	1, 92	32.633	<0.001
	Time	2, 46	17.597	<0.001
	Treatment × time	2, 92	0.831	0.443
TRP	Treatment	1, 92	743.140	<0.001
	Time	2, 46	19.034	<0.001
	Treatment × time	2, 92	11.451	<0.001
fTRP	Treatment	1, 92	234.384	<0.001
	Time	2, 46	35.198	<0.001
	Treatment × time	2, 92	31.166	<0.001
TYR	Treatment	1, 92	1.076	0.311
	Time	2, 46	8.838	0.001
	Treatment × time	2, 92	0.745	0.481
VAL	Treatment	1, 92	8.923	0.007
	Time	2, 46	54.583	<0.001
	Treatment × time	2, 92	1.626	0.208

The within-participant factors in the RMANOVA included ATD/BAL treatment and time (T1–T3); sex was a between-participants factor

### Effects of ATD and time on TRP concentrations, TRP influx across the blood–brain barrier, and the TRP/CAA ratio

Significant main effects of time after AA intake and treatment and a significant treatment-by-time interaction of TRP concentrations and TRP-related parameters were detected (TRP/fTRP plasma levels, TRP/fTRP influx across the blood–brain barrier, the TRP/CAA ratio, and the fTRP/CAA ratio). After ATD ingestion, clear decreases in total TRP and fTRP were observed, while after BAL ingestion, both AAs increased [TRP treatment *F* (1, 92) = 743.140, *p* < 0.001; TRP time *F* (2, 46) = 19.034, *p* < 0.001; fTRP treatment *F* (1, 92) = 234.384, *p* < 0.001; fTRP time *F* (2, 46) = 35.198, *p* < 0.001]. Similar results were identified for the fTRP/CAA ratio [treatment *F* (1, 92) = 187.409, *p* < 0.001; time *F* (2, 46) = 4.514, *p* = 0.016], the TRP/CAA ratio [treatment *F* (1, 92) = 764.177, *p* < 0.001; time *F* (2, 46) = 9.652, *p* < 0.001], total TRP influx [treatment *F* (1, 92) = 1,340.715, *p* < 0.001; time *F* (2, 46) = 3.147, *p* = 0.053] and fTRP influx [treatment *F* (1, 92) = 285.477, *p* < 0.001; time *F* (2, 46) = 4.464, *p* = 0.017]. These results show that these parameters decreased after ATD intake but increased after BAL intake, suggesting that ATD Moja-De lead to a robust reduction in central nervous system TRP availability and 5-HT synthesis.

## Discussion

ATD Moja-De ingestion significantly decreased plasma TRP levels and consequently reduced TRP influx across the blood–brain barrier compared with BAL. This significant reduction in the TRP influx into the brain occurred 90 min after ATD intake and remained stable at this level 180 and 240 min later. Free and total TRP influxes significantly decreased following ATD administration compared with the baseline (85.324 for fTRP at T2 vs. T0 and 81.084 % for TRP at T2 vs. T0, respectively). For ATD compared with BAL, there was a maximum reduction in the TRP influx across the blood–brain barrier, as measured by reduced fTRP levels at T3 (91.305 % under ATD vs. T0) and total TRP levels at T2 (85.900 % under ATD vs. T0). Overall, the data support the assumption that lowering cerebral TRP availability using ATD Moja-De is a reliable and tolerable method of studying CNS serotonin function. This effect is likely due to Moja-De’s composition of different AA quantities and its weight-adapted test protocol. ATD Moja-De promises to be a useful tool in additional investigations of serotonin function in animal, adult, and child studies. Our findings indicate that ATD Moja-De is associated with lower plasma TRP concentrations (both free and total TRP), which accords with the pilot work conducted by Kewitz (Kewitz 2002; Demisch et al. 2002). Because the present study used indirect methods to estimate a decrease in central nervous TRP availability, the Moja-De ATD protocol needs to be validated in animal studies, which is the subject of ongoing research (Biskup et al. 2012).

One major advantage of the present study is that it provides evidence that ATD Moja-De decreases central nervous serotonin function in humans. This is of relevance as ATD Moja-De so far is the only ATD protocol that can be administered to children and adolescents. Following this, the present findings support the use of ATD Moja-De in youngsters, allowing to probe the effects of a short-term decrease in central nervous 5-HT synthesis in youths with different neuropsychiatric disorders as well as healthy subjects. Moreover, as can be seen from the plasma data obtained both total and free TRP decreased significantly under influence of ATD Moja-De, supporting the use of total TRP concentrations in plasma as an estimate for the decrease in central nervous TRP availability after ingestion of ATD Moja-De. This is of relevance, in particular as the determination of free TRP in plasma may cause significant additional costs. The findings of the present study also need to be seen in the light of some limitations. First, although the use of Michaelis–Menten kinetics is a well-established procedure (with evidence coming from multiple animal and human studies), in order to perform calculations as done in the present work this particular approach still provides a somewhat indirect method to estimate a decrease in central nervous 5-HT synthesis in humans. Further studies, for example using positron emission tomography (PET) in combination with ATD Moja-De would be of particular relevance in order to provide reliable data with respect to changes in receptor binding potentials related to serotonergic neurotransmission under influence of ATD Moja-De. Such data would also provide valuable information with respect to brain areas affected by ATD Moja-De. Second, data on potentially induced oxidative stress under influence of ATD Moja-De, in particular after prolonged depletion (for example several days), are lacking, and which should be the subject of future research.

The validity of ATD has been recently questioned in a review article, although the authors of this review did not deny the fact that ATD lead to lowered central nervous 5-HT synthesis (van Donkelaar et al. 2011). One argument raised was that ATD might also involve, or at least lead to, nonserotonergic mechanisms, such as the effects of stress (van Donkelaar et al. 2009b), metabolic and cerebrovascular effects, and, in particular, a changed cerebral blood flow (CBF). For example, studies on altered object memory under ATD in rats showed an improvement after the inhibition of phosphodiesterase (PDE), which in turn can affect CBF, memory, and cognition-related processes (Prickaerts et al. 2002a, b). In addition, ATD as indexed by a diminished TRP/CAA ratio may inhibit nitric oxide synthase (NOS), with evidence obtained from studies on rats (Blokland et al. 1998; Prickaerts et al. 2002a). As a consequence, subsequently diminished citrulline can lead to a reduced nitric oxide (NO) synthesis (Dawson and Dawson 1996; Lieben et al. 2004; van Donkelaar et al. 2009a). This can directly affect CBF.

A moderate rise in NO concentrations leads to diminished 5-HT release, in contrast to an increased 5-HT release after a minor rise in NO levels (Kaehler et al. 1999; van Donkelaar et al. 2011). In addition, the diminished activity of different phosphodiesterase (PDE) isoenzymes (PDE5-I, PDE2-I) facilitates NOS activity, affecting cognition and memory through the outlined pathway (Kelly et al. 1994, 1995; Blokland et al. 1998; Prickaerts et al. 2002a). PDE5-I and PDE2-I facilitate levels of second messengers, such as cyclic guanosine monophosphate (cGMP) and cyclic adenosine monophosphate (cAMP), with cGMP and cAMP having an impact on memory and learning (van Donkelaar et al. 2011; Domek-Lopacinska and Strosznajder 2008; Murad et al. 1978). However, to date, evidence supporting these mechanisms in relation to ATD in humans is lacking. Moreover, as stated by Crockett et al. (2011), there is sufficient evidence indicating that ATD clearly diminishes stimulated 5-HT release and, above all, brain 5-HT. Further secondary mechanisms related to ATD, in particular processes that can affect CBF, need to be taken into consideration and should be the subject of further investigation.

CBF is also known to impact the ratio of free TRP to albumin-bound TRP. As outlined by van Donkelaar et al. (2011), the interaction between the glycocalix of the blood–brain barrier and albumin-bound TRP can be increased by low CBF, resulting in a higher dissociation of TRP from albumin (Pardridge and Fierer 1990; Smith et al. 1987b; van Donkelaar et al. 2011). There is a notable debate over whether free or total TRP reflects the reduced influx of TRP over the blood–brain barrier most accurately. On the one hand, data obtained in rats suggest a positive correlation between free TRP and TRP concentrations in the whole brain (Oldendorf and Szabo 1976; Biggio et al. 1974). Moreover, the dissociation of albumin-bound TRP induced by endogenous and exogenous ligands resulted in a stronger influx of TRP into the CNS (van Donkelaar et al. 2011; Gessa and Tagliamonte 1974; Tagliamonte et al. 1973). On the other hand, there is also evidence supporting the hypothesis that total TRP concentrations represent a better indicator for the decreased influx of TRP into the brain after ATD administration, which is partly in line with the present results. In particular, TRP is known to be loosely bound to albumin, and albumin was shown to undergo significant conformational changes, although it cannot enter the CNS alone (van Donkelaar et al. 2011; Reed and Burrington 1989). In turn, as outlined by van Donkelaar et al. (2009a, b, 2011), the reported conformational changes support a dissociation of TRP from albumin in the cerebral microvasculature and depend on cerebral haemodynamical processes (Pardridge and Fierer 1990). Furthermore, an altered CBF can affect the interaction between albumin-bound TRP and the blood–brain barrier glycocalix with a diminished CBF leading to a higher dissociation rate of TRP from albumin (van Donkelaar et al. 2011; Pardridge and Fierer 1990; Smith et al. 1987b).

As shown by the present data, the ATD Moja-De paradigm leads to a significant reduction in both the fTRP and TRP concentrations, suggesting that this particular ATD protocol leads to a robust reduction in the uptake of TRP into the CNS and subsequently, to a lowered 5-HT synthesis rate (see Figs. 1a, b, 2a, b). In conclusion, as outlined by van Donkelaar et al. (2011), spatial as well as temporal dynamic changes in CBF can impact TRP uptake into the brain. This bears a close relationship to the previously mentioned trials on altered object memory under ATD in rats (Blokland et al. 1998; Prickaerts et al. 2002a), suggesting that NOS, NO and PDE isoenzymes play a decisive role in these parameters with respect to ATD-induced effects, which are not solely dependent on a diminished 5-HT synthesis rate, in particular when memory and cognition are concerned (Kelly et al. 1994, 1995).

The discovery that ATD Moja-De decreases TRP influx across the blood–brain barrier could also be of clinical value. For example, future research could aim to probe the susceptibility to an acute central nervous system 5-HT dysfunction as achieved by ATD and examine the physiological, affective and behavioral responses under ATD as a potential outcome predictor, e.g., as regards treatment with SSRIs in patients with depression and other mood disorders, as well as eating disorders. However, at this stage, ATD Moja-De must be considered as a primarily experimental paradigm to probe the effects of a short-term reduction in central nervous 5-HT synthesis, and the value of ATD Moja-De as a clinical neurodietary challenge test in vulnerable populations needs to be confirmed by future investigations.

In summary, the present investigation indicates that ATD Moja-De effectively diminishes TRP influx into the brain and CNS 5-HT synthesis in healthy adults, as measured by a decreased substrate availability for central nervous 5-HT synthesis. Future studies with larger samples of both healthy humans and patients with neuropsychiatric disorders related to changes in serotonergic neurotransmission might illuminate the underlying relationship between the central nervous availability of 5-HT and changes in neurotransmission in neuropsychiatric disorders and other phenotypes related to mental illness. The Moja-De ATD paradigm allows such research to be undertaken from a developmental viewpoint because it effectively decreases TRP influx into the brain and can be administered to children and adolescents.

## Electronic supplementary material

Below is the link to the electronic supplementary material.

Supplementary material 1 (PDF 106 kb)
