# Supplementary material for: Acute tryptophan depletion in accordance with body weight: influx of amino acids across the blood–brain barrier
Source: J Neural Transm (Vienna). 2012 May 24;119(9):1037–45. doi: 10.1007/s00702-012-0793-z (PMC3424286; doi:10.1007/s00702-012-0793-z)
Supplement: Supplementary file 1 — Supplementary material 1 (PDF 106 kb) [file 702_2012_793_MOESM1_ESM.pdf]

Supplementary online materials

Tab. 2

	Age (years)	Weight (kg)	BMI (kg/m^2)
**Full sample**	25.34 ± 2.09	70.54 ±11.86	23.04 ± 1.86
**Males**	25.39 ± 2.43	80.08 ± 6.30	24.01 ± 1.55
**Females**	25.30 ± 1.69	61.00 ± 7.70	22.07 ± 1.63

Legend: Tab. 2 provides the means ± standard deviations of the characteristics of the full sample (n = 24) and for males (n = 12) and females (n = 12).

Tab. 3

		ATD								BAL							
Amino acid	Sample	Mean T0	SD T0	Mean T1	SD T1	Mean T2	SD T2	Mean T3	SD T3	Mean T0	SD T0	Mean T1	SD T1	Mean T2	SD T2	Mean T3	SD T3
ILE	m	67.6	12.1	337.0	95.0	346.6	69.2	166.4	56.9	66.9	16.8	254.4	61.5	293.0	77.4	189.8	68.8
	f	63.5	14.4	273.3	96.8	303.1	118.0	212.6	89.1	60.5	14.3	256.7	85.9	292.9	106.2	221.8	127.3
	Total	65.6	13.2	305.2	99.3	324.8	97.1	189.5	76.8	63.7	15.6	255.6	73.1	293.0	90.9	205.8	101.4
LEU	m	131.0	22.0	582.9	121.4	560.2	119.1	295.4	93.6	130.4	31.2	501.8	96.3	506.1	102.2	298.7	107.2
	f	113.0	26.2	534.1	136.4	535.0	203.0	310.1	114.0	110.5	27.7	495.2	102.3	497.8	149.6	321.5	161.9
	Total	122.0	25.4	558.5	128.7	547.6	163.3	302.7	102.3	120.5	30.6	498.5	97.3	502.0	125.3	310.1	134.8
LYS	m	155.0	32.0	536.1	86.0	434.7	84.8	275.9	53.0	150.4	30.7	471.4	97.4	351.8	43.7	235.3	61.5
	f	139.2	44.7	563.9	135.5	418.5	135.9	258.7	65.5	137.2	29.7	490.0	102.9	350.9	85.3	220.9	61.8
	Total	147.1	38.9	550.0	111.8	426.6	111.1	267.3	58.9	143.8	30.3	480.7	98.5	351.3	66.3	228.1	60.7
MET	m	28.5	6.8	209.5	49.4	239.7	58.4	167.3	44.0	25.8	5.8	183.2	39.4	198.9	35.5	150.3	41.3
	f	24.1	6.1	214.9	47.1	252.1	57.4	200.9	46.5	25.7	5.3	209.2	44.0	231.1	58.3	190.2	58.4
	Total	26.3	6.7	212.2	47.3	245.9	57.0	184.1	47.5	25.7	5.5	196.2	43.0	215.0	50.0	170.3	53.5
PHE	m	61.3	7.5	404.4	76.3	461.8	98.3	288.5	88.8	63.5	9.9	354.8	67.4	393.4	87.1	270.3	67,5
	f	53.6	8.0	444.6	87.2	542.9	146.9	379.2	114.2	57.5	10.0	443.4	116.3	490.7	134.8	356.9	73.4
	Total	57.5	8.5	424.5	82.7	502.3	129.1	333.9	110.2	60.5	10.2	399.1	103.4	442.1	121.6	313.6	81.9
THR	m	125.7	16.9	277.7	62.5	279.8	48.4	229.0	38.6	126.1	26.1	243.9	45.5	222.2	21.1	185.6	35.9
	f	146.5	43.8	309.5	69.2	306.3	59.1	272.3	75.3	146.0	43.6	269.6	57.3	259.8	63.2	232.1	78.2
	Total	136.1	34.1	293.6	66.5	293.0	54.5	250.7	62.6	136.0	36.6	256.8	52.3	241.0	49.9	208.8	64.1
TYR	m	62.8	14.5	109.2	19.6	113.1	27.5	95.9	25.2	59.3	11.8	103.4	28.4	96.8	27.9	81.9	22.5
	f	53.3	13.4	88.1	16.9	94.0	17.9	89.5	13.3	54.6	15.7	90.7	29.1	95.1	41.2	90.7	39.6
	Total	58.0	14.5	98.6	20.9	103.5	24.7	92.7	20.0	57.0	13.8	97.0	28.9	95.9	34.4	86.3	31.8
VAL	m	219.8	40.0	912.3	150.1	931.7	156.1	609.0	157.5	218.9	44.2	818.7	126.1	808.8	105.8	572.1	131.3
	f	197.3	42.6	932.9	168.1	948.9	226.8	657.4	140.5	197.9	42.4	911.0	114.4	891.4	201.3	666.0	200.6
	Total	208.6	42.0	922.6	156.2	940.3	190.6	633.2	148.0	208.4	43.7	864.8	126.8	850.1	162.8	619.0	172.6
TRP	m	55.3	11.0	49.8	16.8	37.7	21.5	29.0	16.4	56.0	11.6	336.5	53.4	368.5	66.2	283.8	71.8
	f	54.4	7.9	53.8	20.9	39.9	25.7	28.4	18.0	56.1	9.9	355.2	48.9	395.2	85.1	318.2	81.4
	Total	54.8	9.4	51.8	18.6	38.8	23.2	28.7	16.8	56.0	10.5	345.9	51.0	381.8	75.8	301.0	77.1
fTRP	m	11.3	3.1	10.8	4.8	8.0	4.4	5.9	3.6	11.4	3.9	105.1	28.5	132.1	36.3	79.6	37.8
	f	10.9	3.0	11.4	4.9	8.0	5.4	5.9	3.8	12.1	3.9	126.0	41.0	153.5	46.4	100.0	37.8
	Total	11.1	3.0	11.1	4.8	8.0	4.8	5.9	3.6	11.7	3.8	115.5	36.1	142.8	42.2	89.8	38.4

Legend: Tab. 3 provides concentrations (µmol/l) of the amino acids isoleucine (ILE), leucine (LEU), lysine (LYS), methionine (MET), phenylalanine (PHE), threonine (THR), tyrosine (TYR), valine (VAL), total (TRP) and free tryptophan (fTRP) obtained under acute tryptophan depletion (ATD) and after the intake of a TRP balanced amino acid load (BAL), which served as a control condition. The data were obtained at different time points (T0-T3); T0 served as a baseline prior to ATD/BAL administration on separate days (T1 = 90 minutes, T2 = 180 minutes, and T3 = 270 minutes after ATD/BAL intake). The values are depicted as the means ± standard deviations (SDs) for the full sample (total) and for females (f) and males (m).
